# High-resolution structure of the amino acid transporter AdiC reveals insights into the role of water molecules and networks in oligomerization and substrate binding

**DOI:** 10.1186/s12915-021-01102-4

**Published:** 2021-08-30

**Authors:** Hüseyin Ilgü, Jean-Marc Jeckelmann, David Kalbermatter, Zöhre Ucurum, Thomas Lemmin, Dimitrios Fotiadis

**Affiliations:** 1 Institute of Biochemistry and Molecular Medicine, and Swiss National Centre of Competence in Research (NCCR) TransCure, University of Bern, CH-3012 Bern, Switzerland; 2 DS3Lab, System Group, Department of Computer Sciences, ETH Zurich, CH-8093 Zürich, Switzerland; 3 Trkola Group, Institute for Medical Virology, University of Zurich, CH-8057 Zürich, Switzerland

**Keywords:** AdiC, Amino acid transporter, Crystal structure, Membrane protein, Molecular dynamics simulations, Water networks

## Abstract

**Background:**

The L-arginine/agmatine transporter AdiC is part of the arginine-dependent extreme acid resistance system of the bacterium *Escherichia coli* and its pathogenic varieties such as strain *E. coli* O157:H7. At the present time, there is a lack of knowledge concerning the role of water molecules and networks for the structure and function of AdiC, and solute transporters in general.

**Results:**

The structure of the L-arginine/agmatine transporter AdiC was determined at 1.7 Å resolution by X-ray crystallography. This high resolution allowed for the identification of numerous water molecules buried in the structure. In combination with molecular dynamics (MD) simulations, we demonstrate that water molecules play an important role for stabilizing the protein and key residues, and act as placeholders for atoms of the AdiC substrates L-arginine and agmatine. MD simulations unveiled flexibility and restrained mobility of gating residues W202 and W293, respectively. Furthermore, a water-filled cavity was identified at the dimer interface of AdiC. The two monomers formed bridging interactions through water-mediated hydrogen bonds. The accessibility and presence of water molecules in this cavity was confirmed with MD simulations. Point mutations disrupting the interfacial water network validated the importance of water molecules for dimer stabilization.

**Conclusions:**

This work gives new insights into the role and importance of water molecules in the L-arginine/agmatine transporter AdiC for protein stabilization and substrate-binding site shaping and as placeholders of substrate atoms. Furthermore, and based on the observed flexibility and restrained mobility of gating residues, a mechanistic role of the gate flexibility in the transport cycle was proposed. Finally, we identified a water-filled cavity at the dimeric interface that contributes to the stability of the amino acid transporter oligomer.

**Supplementary Information:**

The online version contains supplementary material available at 10.1186/s12915-021-01102-4.

## Background

Liquid water is essential for life on earth since it is the medium in which biological processes take place. In living cells, water plays an active role over different time scales and distances, for example in the transmission of information [1, 2]. As an integral part of biomolecules, in particular of proteins, water governs their structure, stability, dynamics, and function [3]. However, the role of water for the function of biological macromolecules has been underestimated in the past, and still today, most of the literature neglects the central role of water in life’s machineries [1, 2]. From a structural biologist’s point of view, this is understandable, because the visualization of the hydration sites in macromolecular structures requires experimental data at high resolution, i.e., structures at resolutions better than 2.3 Å [4]. Furthermore, the number of observed water molecules will increase with the resolution at which the structure has been solved [5]. For crystal structures of soluble proteins, resolutions at < 2.3 Å are frequent, which is in stark contrast to membrane proteins where high-resolution structures (and structures in general) are relatively sparse.

The membrane transport protein AdiC is part of the arginine-dependent acid resistance system of *Escherichia coli* [6]. Such resistance systems enable pathogenic enterobacteria, e.g., *E. coli* strain O157:H7, to survive the strong acidity of the stomach and to subsequently infect and colonize the human gut. The L-arginine/agmatine transporter AdiC belongs to the amino acid/polyamine/organocation (APC) transporter superfamily [7, 8] and is a prokaryotic member of the SLC7 family of solute carriers [9]. AdiC forms a homodimer in detergent micelles and lipid membranes [8, 10], and each monomer is a self-contained transporter [11]. At the molecular level, the arginine-dependent acid resistance system of *E. coli* relies on the consumption of intracellular protons through the decarboxylation of L-arginine to agmatine to maintain a pH conducive to cell survival. The produced agmatine is exchanged for external L-arginine through AdiC to provide new amino acid. Functional studies have shown that AdiC is an obligate exchanger, coupling influx of L-arginine with efflux of agmatine across the lipid bilayer [8, 10]. Transporters such as AdiC undergo different conformational states during substrate translocation following the alternating access mechanism (Additional file 1: Fig. S1) [9, 12, 13]. In the translocation cycle, two thin gates prevent bound substrate to diffuse away from the transporter (states c, d, and e in Additional file 1: Fig. S1). A tryptophan residue (W202) was found in AdiC to serve as an outward-facing thin gate (also referred to as proximal gate [14]), preventing diffusion of bound substrate back into the periplasm (state c in Additional file 1: Fig. S1) [14–16]. In outward-open, substrate-bound [15, 16], and outward-facing, substrate occluded AdiC structures (states b and c in Additional file 1: Fig. S1) [14], a second tryptophan residue (W293) was shown to interact via cation-π interactions with L-arginine and agmatine in the substrate-binding pocket. In a model for AdiC-mediated L-arginine/agmatine exchange, W293 was proposed as middle gate [14].

AdiC structures were previously reported at resolutions of 3.6 Å [17], 3.2 Å [11], 3 Å [14, 15], 2.6 Å [16], and 2.2 Å [16]. In the latter two structures, due to the higher resolutions, water molecules could be resolved and identified giving first insights into the role of water in the substrate-binding site [16]. Theoretical studies have proposed important water mediated interactions for the transport of L-arginine through AdiC [18, 19].

In this study, we present the structure of the L-arginine/agmatine transporter AdiC in the outward-open substrate-free state at the unprecedented resolution of 1.7 Å. Such high-resolution structures of membrane transporters and of membrane proteins in general are sparse, but essential for the visualization and identification of functional water molecules and complex water networks. Furthermore, in combination with molecular dynamics simulations, we were able to identify and characterize the dynamics of several important water molecules and networks. In particular, our results provided insights into the role of water molecules in the shaping of the substrate-binding site, as placeholders of substrate atoms, the stabilization of the protein and the dimeric assembly of this transporter.

## Results and discussion

The significant increase in resolution of the AdiC structure was achieved by adapting and adding three steps to our previously published protocol [16]. First, we introduced a size exclusion chromatography (SEC) step before crystallization. Then, crystals were flash-cooled in liquid ethane. Finally, we merged data sets from multiple crystals and applied anisotropic correction to the data (see the “Methods” section).

### Overall structure

**Fig. 1:**
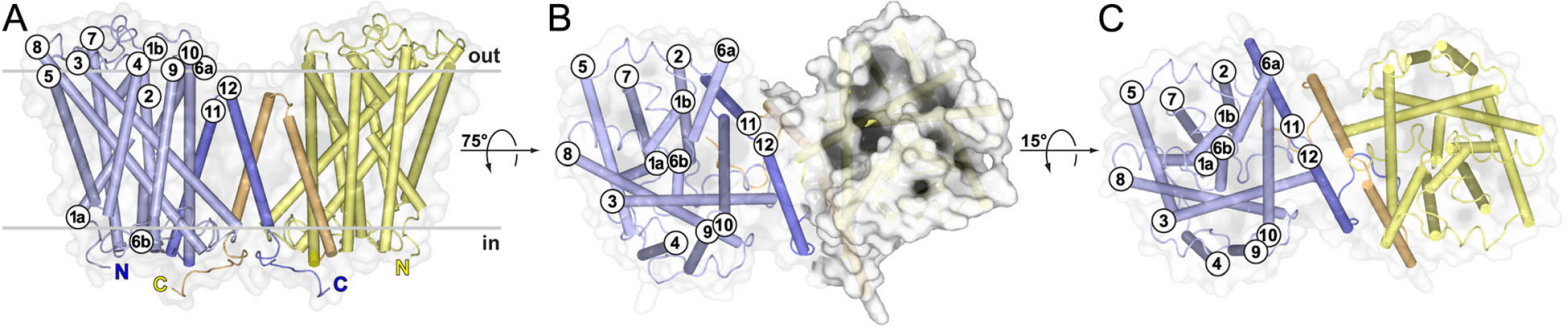
Overall structure of AdiC from *E. coli* in the outward-open conformation. The structure is represented as ribbon and transparent surface, and viewed from the membrane plane (**A**), rotated by 75° (**B**) and from the periplasmic side (**C**). Individual monomers are shown with different shades of blue and yellow. Both termini are located on the cytoplasmic side and are indicated with the corresponding colored capital letters N and C. The twelve TMs of each monomer are represented by cylinders and labeled TM1-TM12. TM1 and TM6 are discontinuous, and each consists of two short α-helices (labeled TM1a and TM1b, and TM6a and TM6b). TMs 1-10 (light blue and yellow) adopt the 5+5 inverted repeats topology and form a barrel-like structure, which is best seen in the surface representation of the right monomer in **B**. TMs 11 and 12 (blue and light orange) constitute most of the homodimer interface

The crystal structure of AdiC from *E. coli* was solved at the unprecedented resolution of 1.7 Å by X-ray crystallography (Additional file 1: Table S1). AdiC is homodimeric and each monomer consists of twelve transmembrane α-helices (TMs) with intracellular N- and C-termini (Fig. 1). The high quality of the obtained electron density map can be assessed in Additional file 1: Fig. S2. The structure described here was obtained in the outward-open state. TM1–TM10 form a barrel-like structure surrounding a relatively large solvent filled cavity, which is exposed to the periplasmic space (Fig. 1B). Ten TMs constitute the core of the protein and adopt the 5+5 inverted repeats topology, which is typical of the APC superfamily [12, 13]. TM1 and TM6 are discontinuous, each forming two short α-helices: TM1a and TM1b, and TM6a and TM6b, respectively (Fig. 1 and Additional file 1: Fig. S3A). These short α-helices are connected by loops, which are known to be involved in substrate-binding [14–16]. The interface of the dimer was calculated with PISA [20] and the surface is equal to 2629 Å^2^. Most of the AdiC homodimer interface is formed by non-polar amino acids from TM11 and TM12, where residues of TM11 from one monomer interdigitate with residues of TM12 from the other monomer (Additional file 1: Fig. S3B). Further interactions between the two monomers are mediated by the loops between TM2 and TM3, the cytoplasmic ends of TM2 and TM3, the cytoplasmic halves of TM10, and the C-termini. The latter embrace neighboring monomers (Fig. 1A, bottom).

The unprecedented high resolution of the AdiC structure allowed for the identification of numerous water molecules and networks. Such structural information is very rare for membrane proteins and offers a unique opportunity to dissect these crystallographic water molecules and networks and directly characterize their dynamics and stability with molecular dynamics (MD) simulations. Therefore, a set of four independent all atom MD simulations was performed under physiological conditions. Each simulation was carried out up to 500 ns. The structure of AdiC remained stable throughout all the simulations (average RMSD: 2.1 ± 0.1 Å). The analyses focused on three important regions of the AdiC transport protein: (i) the substrate-binding site, (ii) the W202 and W293 gates, and (iii) the cavity at the dimeric interface.

### Water shapes and stabilizes the substrate-binding site

**Fig. 2:**
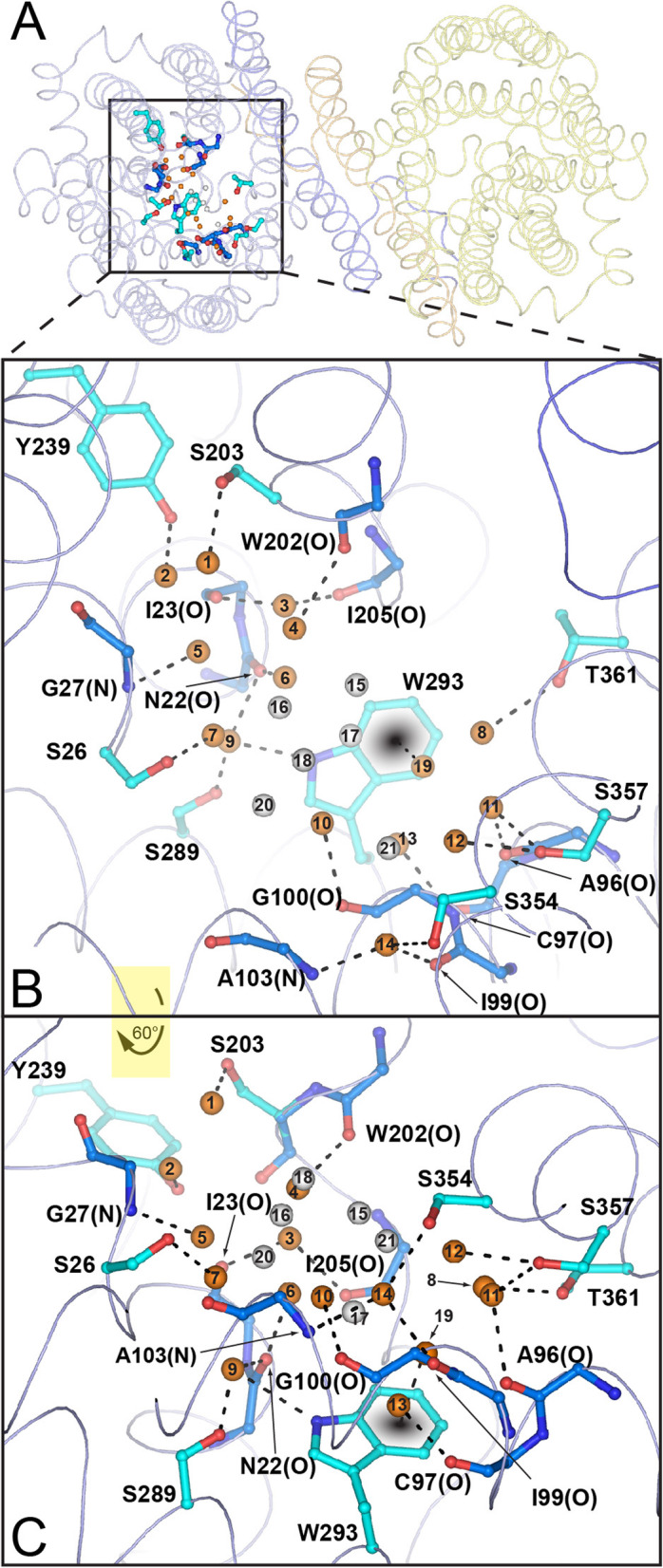
Water molecules in the substrate-binding site. **A** Overall structure of AdiC viewed from the periplasmic side. **B** Enlargement of the box in **A**. Crystallographic bulk (gray spheres) and hydration (orange spheres) water molecules are displayed. Interactions between hydration water molecules with proteinaceous amino acids (distances ≤ 3.2 Å) are shown as dashed lines. **C** Perspective in **B** after ~ 60° rotation of the viewing angle around the *x*-axis. AdiC monomers are represented as transparent ribbons and are colored in blue and yellow. Amino acids are colored in blue (main chain interactions) and cyan (side chain interactions) and are labeled using the one letter code. In addition, main chain interactions with the carbonyl oxygen or nitrogen atom are labeled with (O) or (N)

The substrate-binding site of AdiC has previously been identified and described [14–16]. For comparison purposes, we define the solvent accessible volume between the indole nitrogen atom of W293 and the oxygen atom of the S26 side chain as the substrate-binding site of AdiC in outward-open crystal structures. In the 2.2 Å AdiC structure, seven water molecules were identified in this volume for each protomer [16] (Additional file 1: Fig. S4A). The significant increase in resolution in the presented high-resolution structure, allowed us to identify twenty-one and fifteen water molecules in monomers A and B, respectively (Additional file 1: Fig. S4B). This comparison reflects the important enhancement in water information obtained from the 1.7 Å resolution crystal structure. The water molecules in the substrate-binding site form a water cluster network (Fig. 2). We defined hydration water as water molecules that interact directly with the protein (Fig. 2, orange spheres), and bulk water as water molecules that do not interact with the protein, but only with other water molecules (Fig. 2, gray spheres). This allowed us to discover water molecules that are involved in the shaping and stabilization of the substrate-binding site by linking different TM domains (Fig. S5A and Table S2 in Additional file 1).

### Seven water molecules act as placeholders for substrates atoms

**Fig. 3:**
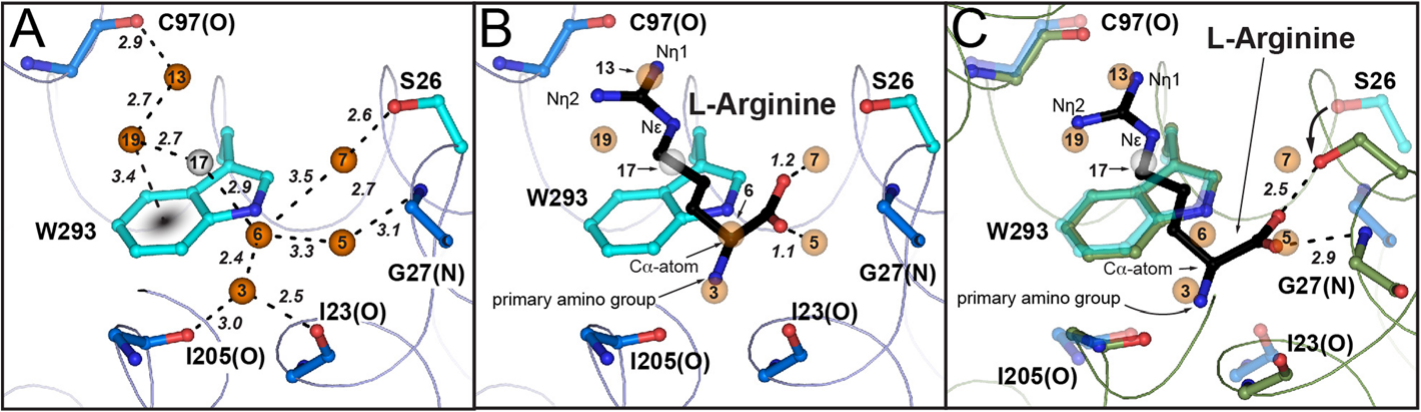
Specific water molecules align with atoms of the AdiC substrates L-arginine and agmatine. **A** Water network in the substrate-binding pocket of the 1.7 Å resolution AdiC structure in the outward-open conformation. **B** Modeled L-arginine in the high-resolution structure based on the agmatine bound outward-open AdiC structure [16]. Water molecules H_2_O3 and H_2_O13 are located in the vicinity of the primary amino and Nη1 guanidinium groups nitrogen atoms of L-arginine or agmatine. Water molecules H_2_O5 and H_2_O7 are located near the modeled oxygen atoms of the L-arginine carboxyl group. **C** Comparison of the outward-open conformation with functional water molecules (**A**) with the L-arginine bound outward-facing, occluded conformation of AdiC-N22A (carbon atoms in green) [14]. Interactions are shown with dashed lines and label in Å. Waters are depicted as orange or gray spheres according to Fig. 2. Amino acids are labeled in the one letter code and when interacting with their main-chain carbonyl oxygen or nitrogen atom additionally labeled with (O) or (N) and colored in blue (**A–C**) and green (**C**), whereas amino acids interacting with the side chains are colored in cyan (**A**–**C**) and green (**C**)

Previously, we showed that two water molecules serve as placeholders for specific agmatine nitrogen atoms in the absence of ligand in the substrate-binding site of AdiC [16]. These water molecules, labeled H_2_O3 and H_2_O13 in Fig. 3A, occupy the nitrogen atoms positions of the primary amino and Nη1 guanidinium groups of agmatine. Due to the achieved high resolution, additional water molecules could be identified in the substrate-binding pocket of the new AdiC crystal structure (Fig. 3A). Based on the modeled L-arginine, the new water molecules H_2_O5 and H_2_O7 are located near the positions expected to be occupied by the two oxygen atoms of the L-arginine carboxyl group in the outward-open, substrate-bound state (Fig. 3B). This finding suggests that H_2_O5 and H_2_O7 replace the oxygen atoms of the L-arginine substrate in its absence. We then compared the modeled L-arginine (Fig. 3B) bound to the outward-open conformation (Additional file 1: Fig. S1, state b) with the L-arginine bound in the outward-facing, occluded AdiC-N22A structure (Additional file 1: Fig. S1, state c) [14]. This comparison revealed that in the outward-facing, occluded conformation, one oxygen of the L-arginine carboxyl group takes the position of H_2_O5, while the second oxygen moves away from the H_2_O7 position (Fig. 3C). In addition, conformational change from the outward-open to the outward-facing, occluded state positions the oxygen atom of the S26 hydroxyl side chain near the position of H_2_O7 (Fig. 3C). H_2_O5 and H_2_O7 are in hydrogen bonding distance to H_2_O6, which would be replaced by the Cα-atom of L-arginine (Fig. 3B) or by the corresponding carbon atom in agmatine. Thus, H_2_O5 and H_2_O7 stabilize the agmatine by weak C–H hydrogen bonds [21]. This observation would explain how the binding site of AdiC can accommodate both, L-arginine and agmatine. H_2_O19 forms a lone pair-π-interaction [22] with the side chain of W293 in the outward-open conformation (Fig. 3A). This interaction is disrupted and H_2_O19 is replaced by Nη2 from the L-arginine guanidinium group in the outward-facing, occluded conformation. Interestingly, this direct interaction with the middle gate residue W293 could represent a potential trigger in the transport mechanism of AdiC. Finally, the water network within the substrate-binding site is completed by the newly identified bulk water molecule H_2_O17, which is in hydrogen bond distance to H_2_O19 and H_2_O6 (Fig. 3A). With the exception of H_2_O13, the MD simulations support the presence of all water molecules acting as substrate atoms placeholders: H_2_O3: 74 ± 9%, H_2_O5: 65 ± 2%, H_2_O6: 33 ± 11%, H_2_O7: 17 ± 2%, H_2_O17: 28 ± 3%, and H_2_O19: 31 ± 12%. In summary, we suggest that binding of L-arginine to AdiC in the outward-open conformation replaces water molecules H_2_O3, H_2_O5, H_2_O6, H_2_O7, H_2_O13, H_2_O17, and H_2_O19 (Fig. 3A). The interchange between these seven structural water molecules and L-arginine has the advantage to preserve the geometry of the binding site in the substrate-free and substrate-bound outward-open states (Additional file 1: Fig. S1, states a and b), thus keeping the energetics in the protein minimal. It is noteworthy that in the L-arginine bound outward-facing, occluded AdiC-N22A structure [14], the position of H_2_O7 is taken by the S26 hydroxyl group, indicating a potential collective behavior between the water molecules, L-arginine, and S26 during the binding event and the transport dependent conformational change.

### Crystallographic water molecules and networks constraining the spatial orientation of the gates W202 and W293 in the outward-open conformation and analyses of their stability with MD simulations

**Fig. 4:**
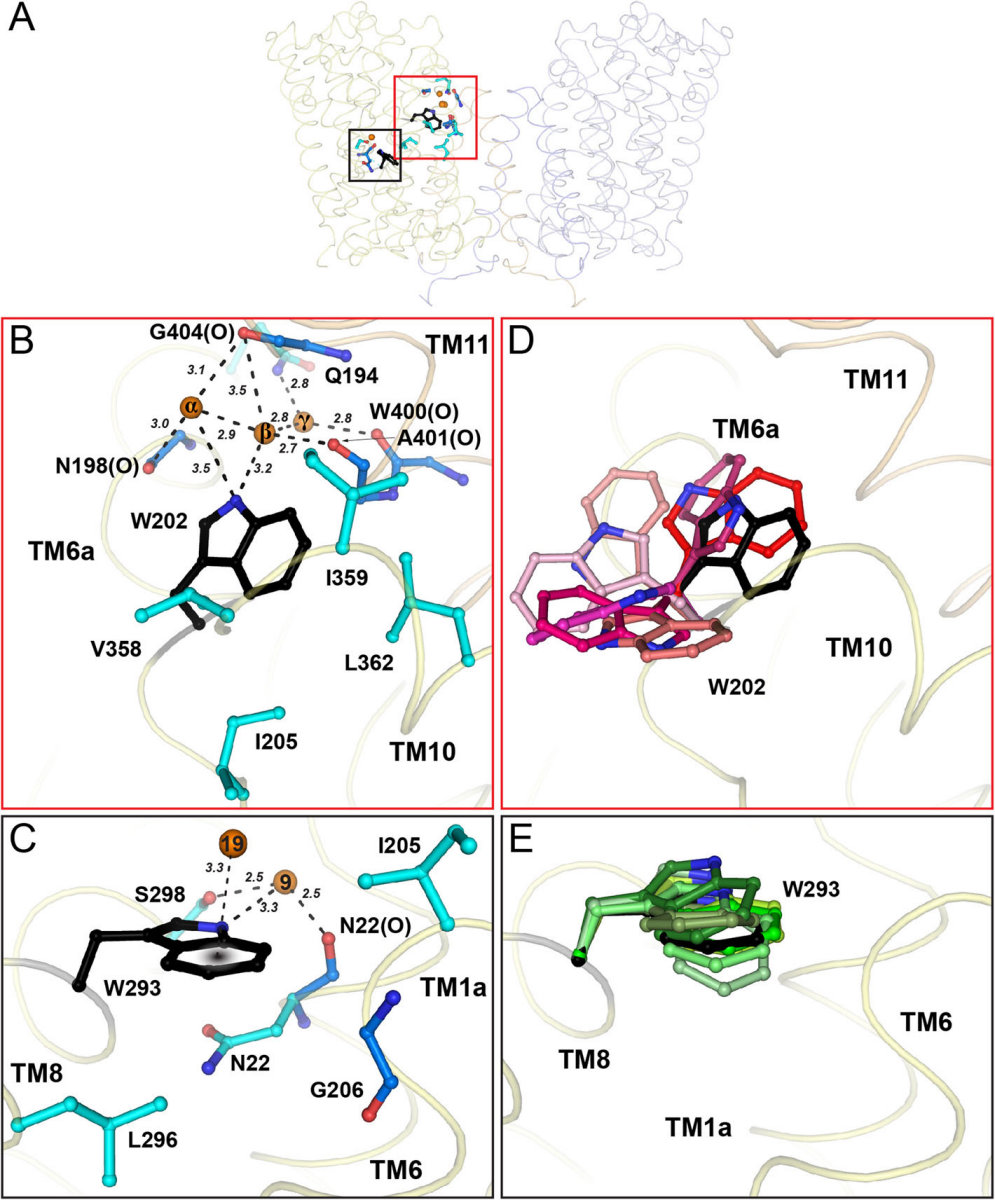
Orientation of gating residues W202 and W293 in the AdiC crystal structure and molecular dynamics (MD) simulations analysis. **A** Side view of the AdiC dimer structure (monomers in yellow and light blue) as seen from the membrane plane. The regions containing the proximal W202 and middle W293 gates are highlighted with a red and black square, respectively. **B** Hydrogen bond interactions observed between the W202 nitrogen atom, three water molecules (α, β and γ), and main and side chain atoms are shown as dashed lines and distances are indicated in Å. Hydrophobic residues (I205, V358, I359, and L362) interacting with W202 within ≤ 4 Å are displayed. **C** Water molecule H_2_O9 mediated hydrogen bonds between the more buried middle gate residue W293, and S298 and N22(O) are shown as dashed lines and distances are indicated in Å. Residues (N22, I205, G206, L296) interacting with W293 within ≤ 4 Å are displayed. Waters are depicted as orange spheres and amino acids are colored in blue (main chain interactions) and cyan (side chain interactions). Amino acids are labeled in the one letter code whereas main chain interactions with the carbonyl oxygen or nitrogen atom are additionally labeled with (O) or (N). **D**, **E** Selected conformations of the W202 (**D**) and W293 (**E**) side chains from MD simulations. W202 and W293 are displayed in red and green hues, respectively. The W202 and W293 conformations found in the crystal structure are colored in black

Conformational changes of AdiC upon binding of L-arginine were suggested [14, 18], which result among other things in the occlusion of the bound substrate from the periplasm by the movement of W202 (transition from states b to c in Additional file 1: Fig. S1). We thus investigated the possible implication of water molecules for the orientation of W202 in the conformation open to the periplasm (state a of the transport cycle in Additional file 1: Fig. S1). The side chain of W202 (TM6a) in the outward-open conformation forms hydrogen bonds with two water molecules: H_2_Oα and H_2_Oβ (Fig. 4A, B). These water molecules interact with N198(O) (TM6a) and G404(O) (TM11) and A401(O) and G404(O) (TM11), respectively. A third water molecule (H_2_Oγ) bridges the amide nitrogen atom of Q194 (TM6a) and W400(O) (TM11). Together, these three water molecules form a string of water at the interface between TM6a and TM11. Importantly, the indole nitrogen atom of the functionally relevant W202 residue does not form direct hydrogen bonds with the protein. Instead, it interacts indirectly with water molecules H_2_Oα and H_2_Oβ, thus adopting a specific spatial orientation. In addition, hydrophobic interactions are observed between W202 and hydrophobic residues in TM6 (I205) and TM10 (V358, I359 and L362) (Fig. 4B). As a consequence, the spatial orientation of the thin gate W202 in the outward-open conformation might be controlled by a water network and hydrophobic side chain interactions. However, in the molecular dynamics (MD) simulations, the observed water network (H_2_Oα-H_2_Oγ) interacting with W202 in the AdiC crystal structure (Fig. 4B) was disrupted, allowing W202 to sample a large range of conformations (Fig. 4D and Additional file 1: Fig. S6). We hypothesize that this water network is most likely only formed during crystallization.

The middle gate residue W293 [14] is located in the substrate-binding pocket of AdiC. In contrast to W202, the spatial orientation of W293 is stabilized by a hydrogen bond to H_2_O9 (Fig. 4C). This crucial water molecule is in turn stabilized through H-bond interactions with N22(O) and S289 (Fig. 4C and Additional file 1: Fig. S5D). W293 is also interacting with the N22 side chain (amide-π interaction) and neighboring hydrophobic residue. The H_2_O9-mediated interaction could help orient W293 in space, which is important since L-arginine and agmatine have indicated cation-π interactions between this tryptophan side chain and the guanidinium groups of the substrates [14–16]. This constrained orientation of W293 is supported by the MD simulations (Fig. 4E and Additional file 1: Fig. S6). The hydrogen bond interactions between H_2_O9 and N22(O), and the nitrogen atom and hydroxyl group of the W293 and S298 side chains is present in about 26 ± 6% of the conformations sampled during MD simulations. The amide-π interaction with N22, either directly or mediated through a water molecule, was more persistent during MD (43 ± 18%). This finding is supported by the N22A mutation, which locks AdiC in the outward-facing occluded state [14].

### Potential mechanistic importance of gate flexibility in the transport cycle

**Fig. 5:**
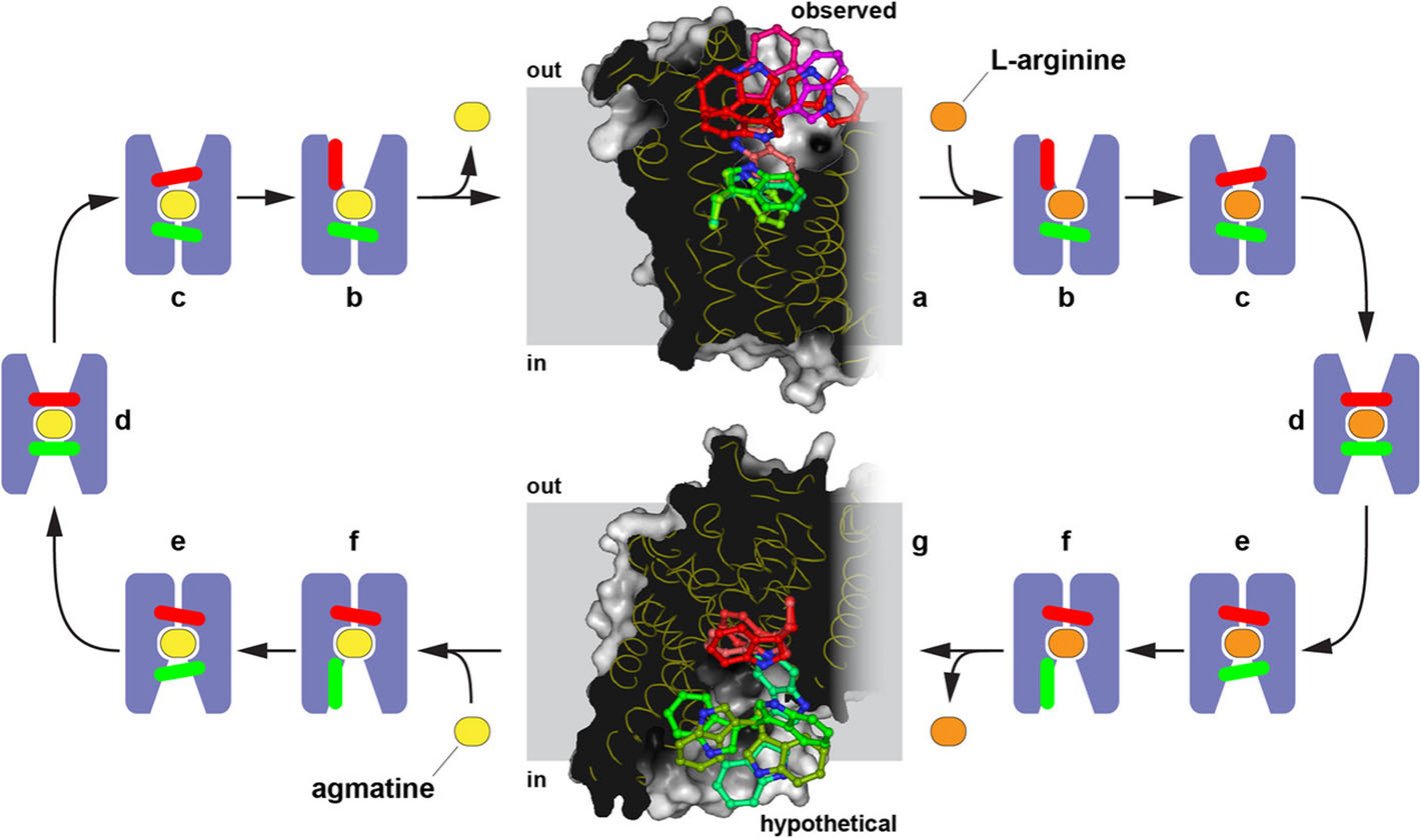
Refined transport cycle of AdiC showing different conformational states and gating residue movements during substrate translocation. In the outward-open, substrate-free state (state a, observed), the gating residue W202 (in red) is mobile, whereas the mobility of the buried gating residue W293 (in green) is restrained. In the inward-open, substrate-free state (state g, hypothetical), the features of the gating residues are inverted, i.e., W293 mobile and W202 restrained mobility. For states a and g, AdiC monomer models are displayed as gray volume with vertical cut through the structure. The membrane region is displayed as gray bar and the sidedness indicated. Gating residues W202 and W293 are shown as red and green sticks, respectively. For states b to f, AdiC monomers are displayed in blue, and gating residues W202 and W293 are shown as red and green bars. The substrates L-arginine and agmatine are colored in orange and yellow, respectively. AdiC conformational states are outward-open, substrate-free (a); outward-open, substrate-bound (b); outward-facing, substrate occluded (c); substrate-bound, fully occluded (d); inward-facing, substrate occluded (e); inward-open, substrate-bound (f); and inward-open, substrate-free (g)

Based on the MD simulations performed using the here presented high-resolution crystal structure of AdiC in the outward-open state, we found that the gate W202 is mobile adopting different conformations (Fig. 4D). In contrast, the mobility of gate W293 is constrained in this state (Fig. 4E). In a previous study [18], a transient cation-π interaction of L-arginine with W202 was proposed, before binding to W293 in the substrate-binding site. Together with our finding on the mobility of W202 in the outward-open state, we propose that W202 might screen for substrate. Once L-arginine is transiently bound to W202, the substrate would slide into the substrate-binding site, where it interacts via cation-π interaction with W293 and hydrogen bonds with other amino acids. This mechanism might be extrapolated to the inward-open conformation, i.e., W293 would be mobile looking for substrate, in this case agmatine, while W202’s mobility would be restrained—see Fig. 5 for a summary and a refined transport cycle. This mechanistic insight from the AdiC gate residues W202 and W293 might be expanded to AdiC homologs of enteropathogens as well as the amino acid-diamine transporters PotE [23] and CadB [24] from *E. coli*. In such members of the amino acid/polyamine/organocation (APC) transporter superfamily [7, 8], tryptophan residues corresponding to the W202 and W293 gates in AdiC are found at corresponding positions in their amino acid sequences (Additional file 1: Fig. S7).

### Water containing cavity at the dimer interface

We identified a heart-shaped cavity at the interface of the AdiC dimer that is lined by mostly hydrophobic amino acids, F84, L85, V363, L366, F414, L417, and M418, and a few polar groups, Q88, Y367, and T421 (Additional file 1: Fig. S8). The high-resolution structure allowed us to detect a residual positive Fo-Fc density in this cavity during protein model refinement (Additional file 1: Fig. S9A). Solutes from the protein buffer and mother liquor, i.e., Tris(hydroxymethyl)aminomethan (Tris), Polyethylenglycol 400 (PEG400), and *n*-nonyl-β-D-glucopyranoside (see the “Methods” section), were too large to fit into the relatively small positive Fo-Fc density. Therefore, we hypothesized the presence of a dynamic interfacial water network and placed water molecules into these Fo-Fc densities during refinement (Additional file 1: Fig. S9A, B). This resulted in the identification of eleven water molecules in the interdimeric cavity (Additional file 1: Fig. S9C). The distances between the water molecules ranged from 1.9 to 3.8 Å. The lower limit is too short to be within hydrogen bond distance, and therefore, not all positions can be occupied simultaneously, supporting our hypothesis of a dynamic water network (Additional file 1: Fig. S9D). Since the data set used for the high-resolution structure determination was derived from twenty-two individual data sets, the observed water network would represent an ensemble of possible positions of water molecules in the cavity. We investigated the hydration of the cavity using the set of MD simulations and identified the positions with the highest probabilities of occupancy. All simulations started with an unsolvated cavity and, within the first 100 ns, water molecules started diffusing into the cavity, mostly from the ligand-binding pocket. The cavity remained hydrated throughout the simulation. On average, the cavity was occupied by approximately 2 water molecules, with the following frequencies for the main configurations: one (20%), two (38%), three (29%), and four (9%) water molecules. We observed that water molecules preferably occupied positions a-d and only rarely positions e–k (Additional file 1: Fig. S9E, F). This was further supported by the better-defined electron densities observed during refinement for positions a–d (Additional file 1: Fig. S9A, B). Since the distances between water molecules a-d are between 1.9 and 2.2 Å, they cannot exist simultaneously. During the MD simulations, water molecules were observed at different positions and frequencies (Additional file 1: Fig. S10). The interfacial water networks composed of three molecules were mainly localized in the continuous density defined by water molecules a–d (Additional file 1: Fig. S9E). However, they occupied intermediate positions in order to accommodate the hydrogen-bonding distance between them.

**Fig. 6:**
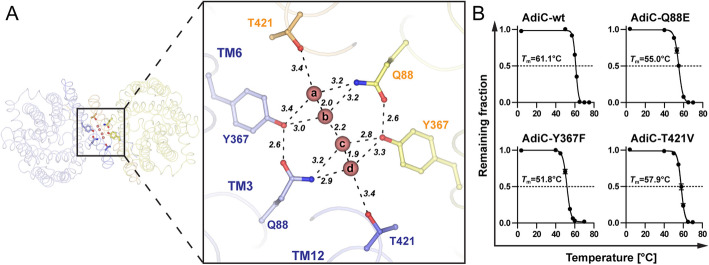
Contribution of water in AdiC dimer stabilization. **A** On the left, the overall structure of AdiC is shown as viewed from the periplasmic side. The small box indicates the location of the cavity at the dimer interface and is displayed enlarged in the right box. All possible hydrogen bond interactions ≤ 3.4 Å are shown as dashed lines and distances are given in Å. The AdiC dimer (transparent ribbons), amino acid side chains involved in water-mediated interface stabilization (sticks), and the labels are colored according to the individual monomeric color code (blue and yellow color hues) as in Fig. 1. Water molecules (a–d) are displayed as red spheres. **B** Comparison of the melting temperature (*T*_m_) of AdiC-wt and three point mutants (AdiC-Q88E, AdiC-Y367F, AdiC-T421V). The determined *T*_m_ values are from at least three independent experiments, each in duplicates. Error bars represent SEM. If not visible, error bars are smaller than symbols

Noteworthy is that the water molecules at positions a–d formed hydrogen bonds with the hydrophilic side chains in the cavity (Q88, Y367 and T421), thus bridging monomers A and B (Fig. 6). This suggests that water molecules are involved in the stabilization of the AdiC dimer. To test this hypothesis, thermostability assays were performed to compare melting temperatures (*T*_m_) between wild-type AdiC (AdiC-wt) and three mutants that would disrupt the water network (Q88E, Y367F and T421V) (Fig. 6B). All mutants eluted at similar volumes as wild-type AdiC during SEC indicating retention of the dimeric state after mutagenesis (Additional file 1: Fig. S11). The mutation Q88E replaces the nitrogen of the amide group by an oxygen atom, which precludes the side chain to serve as a proton donor. With p*K*a values of 3.8 and 4.2 calculated using ROSIE [25] and DelPhiPKa [26], the carboxyl group of the introduced glutamate side chain is deprotonated at physiological pH. The mutations Y367F and T421V replace the side chain hydroxyl groups by a proton or a methyl group, which impedes the formation of hydrogen bonds. In all cases, the thermostability of AdiC was significantly reduced, supporting the importance of water for dimer stabilization. The most drastic change was observed for AdiC-Y367F with a remarkable difference of almost 10 °C (Fig. 6B). For this mutant, the missing interaction of the hydroxyl group of Y367 with the amide group of Q88 (Fig. 6A) in AdiC-Y367F probably also contributed to the dimer destabilization (Fig. 6B).

## Conclusions

The role of water in the function and structure of membrane transporters, and membrane proteins in general, has rarely been addressed, since high-resolution structures of this type of proteins are scarce. Here, we presented the 1.7 Å resolution crystal structure of the L-arginine/agmatine transporter AdiC from *E. coli*. This high-resolution structure combined with MD simulations revealed the implication of water molecules in protein stabilization, the orientation and positioning of key residues in the substrate-binding site, and as placeholders for several atoms of substrates. Furthermore, MD simulations unveiled flexibility and restrained mobility of gating residues indicating a potential mechanistic role in AdiC and other transporters from the APC superfamily. Finally, a hydrated cavity at the dimer interface was observed, and we demonstrated that the water molecules play a key role for the stability of the dimeric AdiC assembly. Our results allowed rare insights into the role and dynamics of water networks in a membrane transporter.

## Methods

### Cloning, mutagenesis and overexpression of AdiC variants

The AdiC wild-type gene was cloned from *E. coli* XL1-Blue genomic DNA into the pZUDF21 vector [27] as described previously [16]. The resulting construct translates into a recombinant AdiC protein containing a human rhinovirus 3C protease (HRV3C) cleavage site followed by a deca His-tag at the C-terminus. Three point mutations (Q88E, Y367F, and T421V) were introduced into the AdiC gene using the QuikChange Lightning Multi Site-Directed Mutagenesis Kit (Agilent). For overexpression, constructs of AdiC variants were transformed into BL21(DE3) pLysS *E. coli* cells and grown in Luria Bertani (LB) medium supplemented with 0.1 mg/ml ampicillin at 37 °C. At an OD_600_ between 0.5 and 0.6, protein expression was induced by addition of isopropyl-β-D-thiogalactopyranoside to a final concentration of 0.3 mM. The growth proceeded for 4 h at 37 °C. Finally, cells were harvested by centrifugation (10,000×*g*, 5 min, 4 °C) and resuspended in 20 mM Tris-HCl, pH 8.0, 500 mM NaCl (Lysis buffer).

### Membrane preparation and purification of AdiC variants

For membrane preparation, cells were lysed using a Microfluidizer M-110P (Microfluidics) at 1500 bar (five passages). To remove cell debris, a centrifugation was applied (10,000×*g*, 10 min, 4 °C). The supernatant was collected and subjected to ultracentrifugation (150,000×*g*, 1.5 h, 4 °C). The obtained pellet was homogenized in Lysis buffer and aliquoted into 2 ml volumes corresponding to membranes from 1 l of cell culture. Aliquots were flash frozen in liquid nitrogen and stored at − 80 °C until further use. For crystallization, membranes containing overexpressed AdiC-wt from 1 l bacterial culture were solubilized for 1 h at 4 °C on a rotational shaker in 20 mM Tris-HCl, pH 8.0, 300 mM NaCl, 10% (v/v) glycerol, 4% (w/v) *n*-nonyl-β-D-glucopyranoside (NG) (Glycon Biochemicals GmbH) and a total volume of 7 ml. For melting temperature (*T*_m_) determination experiments, AdiC variants were solubilized as described for crystallization but using 1.5% (w/v) *n*-dodecyl-β-D-maltopyranoside (DDM) instead of NG and for 2 h. Unsolubilized material was removed by ultracentrifugation (100,000×*g*, 15 min, 4 °C). The supernatant was then collected and diluted twofold in 20 mM Tris-HCl, pH 8.0, 300 mM NaCl, 10% (v/v) glycerol, 0.4% (w/v) NG (for crystallization; Buffer NG) or 0.04% (w/v) DDM (for *T*_m_ determination; Buffer DDM) containing 5 mM L-histidine. The diluted solution was incubated with 0.5 ml (bed volume) of pre-equilibrated Ni-NTA Superflow resin (Qiagen) for 2 h at 4 °C on a rotational shaker. The resin-containing solution was transferred into a column, which was then washed three times with 5 ml of Buffer NG or Buffer DDM containing 5 mM L-histidine, and 3 ml of Buffer NG or Buffer DDM by applying pressure on top of the column. For thermostability experiments (see below), the His-tag of AdiC variants was removed on-column overnight on a rotational shaker at 4 °C using 200 μg of HRV3C (BioVision, Milpitas, CA, USA) [28]. On the following day, the sample was eluted by centrifugation (3,000×*g*, 15 s, 4 °C) and incubated with 0.1 ml (bed volume) of pre-equilibrated Ni-NTA Superflow resin for 15 min on a rotational shaker at 4 °C. Afterwards, the sample was collected by centrifugation (3,000×*g*, 15 s, 4 °C) and used for *T*_m_ determination. For crystallization experiments, the protein-bound resin was incubated with Buffer NG containing 200 mM L-histidine for 15 min at 4 °C on a rotational shaker to separate the protein from the resin. The sample was then eluted by centrifugation (3,000×*g*, 15 s, 4 °C) and the His-tag on AdiC was cleaved by incubation with 300 μg of HRV3C on a rotational shaker for 45 min at 4 °C. After incubation with HRV3C, the sample was concentrated in a 50 kDa Amicon cutoff filter (Sigma-Aldrich) and after centrifugation (21,000×*g*, 5 min, 4 °C), applied to size-exclusion chromatography (SEC) for further purification (20 mM Tris-HCl, pH 8.0, 150 mM NaCl, 0.4% (w/v) NG). The peak fractions were collected, pooled, concentrated to 8–12 mg/ml using a 50 kDa Amicon cutoff filter, and ultracentrifuged (200,000×*g*, 10 min, 4 °C) to remove possible aggregates. The concentrated AdiC-wt protein was mixed with 50 mM Tris-HCl, pH 8.5, 35% (v/v) PEG400, 1% (w/v) NG (reservoir solution) at a 1:1 drop ratio and crystallized using the vapor-diffusion sitting-drop technique. Crystals were collected, flash frozen in liquid ethane [29], and stored in liquid nitrogen until X-ray diffraction analysis.

### Structure determination

All datasets of AdiC were collected at the X06SA (PXI) beamline of the Swiss Light Source (SLS; Paul Scherrer Institute, Villigen, Switzerland) using an EIGER 16M detector (Dectris). Twenty-two datasets from different AdiC crystals were indexed and integrated with XDS [30]⁠ and then merged using BLEND [31]⁠ of the CCP4 program suite [32]⁠ without truncation of the resolution. Scaling and averaging of symmetry-related intensities were performed by aP_scale [33] with truncation of the data at the highest resolution along h, k or l axis determined by AIMLESS [34]⁠. Due to the anisotropic nature of the diffraction data the STARANISO⁠ software (http://staraniso.globalphasing.org/) was applied. This program performs an anisotropic cut-off of merged intensity data for Bayesian estimation of structure amplitudes and applies an anisotropic correction to the data. The structure was solved by molecular replacement employing PHASER implemented in the Phenix software suite [35, 36] using the coordinates of the substrate-free outward-open conformation of AdiC (PDB code 5J4I) [16]. The final structure was obtained after several iterative cycles using default setting of phenix.refine including Translation/Libration/Screw (TLS) parameters (TLS groups were automatically assigned using Phenix) [37] and manual model building using COOT [38]. All data collection, processing, and refinement statistics can be found in Additional file 1: Table S1.

### Modeling of L-arginine from agmatine

Applying the align tool implemented in PyMOL (Version 2.3, Schrödinger, LLC), monomers of the agmatine (1-amino-4-guanidinobutan) bound (PDB 5J4N [16]) and the here presented 1.7 Å AdiC crystal structures in the outward-open conformation were structurally aligned (RMSD = 0.217 for 392 Cα-atoms). The proton at the C1 position of agmatine in the L configuration was replaced by a carboxyl group using the builder tool implemented in PyMOL. The carboxyl group of the resulting L-arginine molecule was rotated such that the nitrogen atom of the primary amino group was coplanar with the carboxyl group since most amino acids are found to cluster around such a conformation [39].

### Thermostability analysis of AdiC variants

Thermostabilities of AdiC variants were determined as described previously [40, 41]. To test the effect of single mutations on the thermostability of AdiC, 35 μg of purified protein samples in aliquots (70 μl total volume) was incubated at different temperatures for 10 min using a Labcycler gradient PCR machine (SensoQuest GmbH). In order to remove eventual aggregates, a short centrifugation was applied (18,000×*g*, 30 s, room temperature). The supernatant was then loaded on a Superdex 200 5/150 GL column (GE Healthcare) installed on an ÄKTA Purifier system (GE Healthcare). For each experiment, the column was equilibrated with 1.5 column volumes of SEC-buffer (20 mM Tris-HCl, pH 8.0, 150 mM NaCl, 0.04% (w/v) DDM). The normalization was done by using the untreated sample as reference, which was kept on ice and then analyzed by SEC. For *T*_m_ determination, the peak absorbance values at 280 nm were used. Finally, *T*_m_ values were calculated by applying the Boltzmann sigmoidal equation in Prism5 (GraphPad Software).

### Molecular dynamics simulations

Molecular dynamics (MD) simulations were carried out in order to characterize the water molecules and networks in AdiC under physiological conditions. The AdiC structure was embedded into an 80 × 80 Å palmitoyloleoly phosphatidylcholine (POPC) membrane. The system was then solvated by adding a 17 Å water layer on both sides of the membrane and neutralized with 150 mM NaCl. The final unit cell measured 80 × 80 × 100 Å^3^. This system was assembled using the CHARMM-GUI webserver. The simulation was performed with the CHARMM36 force field [42], including CMAP corrections for the protein. The water molecules were described with the TIP3P water [43] parameterization. The system was first equilibrated with NAMD molecular engine [44]. The periodic electrostatic interactions were computed using particle-mesh Ewald (PME) summation with a grid spacing smaller than 1 Å. Constant temperature was imposed by Langevin dynamics [45] with a damping coefficient of 1.0 ps. Constant pressure of 1 atm was maintained with Langevin piston dynamics [46], a 200 fs decay period and a 50 fs time constant. During equilibration, the backbone atoms were restrained with harmonic restraints. The system was first minimized by 5000 conjugate gradient steps and then equilibrated by using a linear temperature gradient, which heated up the system from 0 to 310 K in 5 ns. An additional 25 ns were carried out after removing all restraints. The length of all bonds involving hydrogen atoms was constrained with the RATTLE algorithm, thus allowing a time step of 2 fs. For the production runs, we switched to ACEMD engine [47]. The equilibrated system was used as the starting points for four independent MD simulations. After 1000 steps of conjugate gradient descent, each simulation was carried out up to 500 ns, representing a cumulative simulation time of 2.0 μs. The trajectories were analyzed with VMD and in-house scripts implemented in tcl [48]. The water occupancy in the cavity was computed using the VMD volutil plug-in. The grid size was set to 1 Å and the results were averaged for all four simulations.

## Supplementary Information

**Additional file 1: Supplementary Figures and Tables. Fig. S1**. Schematic representation of the alternating access mechanism. The major conformational changes and states of the transporter are shown, which are necessary to allow alternating substrate access from either side of the membrane to the substrate-binding site. Transporter, substrate and lipid molecules are colored in blue, orange and light brown, respectively. The different conformational states shown are a: outward-open, substrate-free; b: outward-open, substrate-bound; c: outward-facing, substrate occluded; d: substrate-bound, fully occluded; e: inward-facing, substrate occluded; f: inward-open, substrate-bound; g: inward-open, substrate-free; h: substrate-free, fully occluded. **Fig. S2**. Quality of the electron density of AdiC. All TMs are displayed as viewed from the membrane plane and boxed into TM-groups belonging to inverted repeats (left) and the dimerization interface (right). Starting and ending amino acid residues of corresponding TMs are labeled. The TMs are displayed as sticks (cyan) and the corresponding 2Fo-Fc electron density map is contoured at 1.0 σ and shown as blue colored mesh. **Fig. S3**. Structural details of AdiC. (A) TM1 and TM6 are discontinuous and their loop regions connecting the α-helical segments are in close proximity and involved in substrate-binding. TM1 and TM6 are represented as light blue ribbons and the side chains as lines. The α-helical segments of TM1 (TM1a and TM1b) and TM6 (TM6a and TM6b) are highlighted as cylinders and labeled accordingly. The respective N- and C-terminal residues of these segments are labeled as well. (B) The largest interface contribution for AdiC homodimer formation is the interaction between TM11 and TM12. TM12 of monomer A (blue) and TM11 of monomer B (yellow) are displayed as ribbons. Interdigitating, non-polar amino acid residues involved in dimer formation are shown as sticks and labeled. **Fig. S4**. Comparison of the number of water molecules found in the substrate-binding sites of AdiC structures solved at different resolutions. (A) In the 2.2 Å AdiC structure [16], seven water molecules could be found in the substrate-binding sites of each monomer. (B) In the 1.7 Å AdiC structure of the present study, twenty-one and fifteen water molecules were detected in monomers A and B, respectively. The substrate-binding site cavity was defined as the solvent accessible volume between the indole nitrogen atom of W293 and the side chain oxygen atom of the S26 (borders are indicated with dashed lines and respective amino acids are shown as gray sticks). The two AdiC monomers are represented as ribbons and are colored in blue and yellow color hues. Water molecules are displayed as red spheres. **Fig. S5**. Water induced linkage of TM domains. (A-D) Interactions of water molecules (orange) with amino acid main chains (blue sticks) and side chains (cyan sticks). Respective interactions between atoms are shown with dashed lines (distances are given in Å). The corresponding 2Fo-Fc electron density map of the interaction regions is contoured at 1.0 σ and is displayed with a light blue mesh representation. Involved TMs are indicated, and amino acids are labeled in the one letter code and when interacting with their main-chain carbonyl oxygen or nitrogen atom additionally labeled with (O) or (N). **Fig. S6**. Side chain torsional angles sampled during molecular dynamics simulations. Density plots representing the *χ*_1_ and *χ*_2_ conformations of W202 (A) and W293 (B). The marginal distributions are obtained using a kernel density estimate. The centroids from a k-means clustering are shown with orange triangles. **Fig. S7**. Multiple sequence alignment of AdiC from *E. coli* K12 and the pathogenic *E. coli* strains O104:H4 and O157:H7, AdiC/CadB homologs (hAdiC/hCadB) from other pathogenic enterobacteria and the amino acid-diamine transporters PotE and CadB from *E. coli* K12. Amino acid sequence alignment was performed with Clustal Omega (https://www.ebi.ac.uk/Tools/msa/clustalo/). The UniProt ID or NCBI reference sequence codes are as follows: AdiC *E. coli* K12 (P60061), AdiC *E. coli* O104:H4 (A0A0E0Y6U0), AdiC *E. coli* O157:H7 (P60063), PotE *E. coli* K12 (P0AAF1), CadB *E. coli* K12 (P0AAE8), hAdiC *Salmonella typhi* (P60065), hAdiC *Shigella flexneri* (P60064), hCadB *Vibrio cholerae* (WP_000097425.1) and hAdiC *Yersinia enterocolitica* (WP_174848373.1). Positions with a single, fully conserved residue are indicated by (*), conservation between groups of strongly and weakly similar properties are indicated by (:) and (·), respectively. Color-coding of amino acid residues is based on their physicochemical properties, i.e., small and hydrophobic (red), acidic (blue), basic (magenta) and other (green) amino acid residues. The highly conserved gating residues W202 and W293 are highlighted in bold and yellow, and the N- and C-terminal numbers of the corresponding amino acid sequence stretches are indicated. **Fig. S8**. Cavity at the dimer interface. Two different views into the heart-shaped inter-dimeric cavity are shown, i.e., as viewed from the membrane plane (A) and as viewed from the periplasmic side (B). The top images represent overviews of the overall AdiC structure displayed as transparent surfaces and ribbons. The panels in the middle illustrate a vertical cut through the structure revealing the presence of a buried cavity (highlighted in red). The boxed structural regions are displayed enlarged in the bottom images. In all panels, side chains shaping the cavity are shown as sticks. All structural elements and labels are colored according to the individual monomeric color code, i.e., in blue and yellow. **Fig. S9**. Water inside the interdimeric cavity. (A) Positive residual Fo-Fc electron density map after the first refinement run. (B) After positioning eight waters (a-c, e-h and j), the second refinement run revealed additional positive residual Fo-Fc electron density. (C) 2Fo-Fc density after the third refinement run with all waters positioned (a-k). (D) Distances between water molecules are shown as black dashed lines and are given in Å. (E) Contour map representing the density probability of the water molecules in the cavity calculated from molecular dynamics simulations. The positions of crystallographic water molecules a-k are indicated with yellow crosses. (F) Corresponding wire-frame rendering of the isosurface contoured at 10% probability. In panels (A-D) and (F), the AdiC structure (transparent ribbons), important amino acid side chains (sticks) and the labels are colored according to the individual monomeric color code, i.e., in blue and yellow. Water molecules are represented as red spheres and are labeled with small case letters. Positive Fo-Fc and 2Fo-Fc electron density maps are represented by meshes, colored in green and blue, and contoured at 3.0 and 1.0 σ, respectively. **Fig. S10**. Different possibilities of water binding at the AdiC dimer interface cavity, i.e., binding of one (A-D) or two (E-G) water molecules. In all panels, distances are in Å and shown as black dashed lines. The AdiC structure (transparent ribbons), important amino acid side chains (sticks) and the labels are colored according to the individual monomeric color code, i.e., in blue and yellow. Water molecules are represented as red spheres and are labeled with the small case letters a-d. Corresponding frequencies (%) according to molecular dynamics simulations of water configurations are indicated in the lower, left corners. **Fig. S11**. Size exclusion chromatography (SEC) of AdiC variants. Wild-type AdiC (AdiC-wt), and AdiC mutants Q88E (AdiC-Q88E), Y367F (AdiC-Y367F) and T421V (AdiC-T421V) eluted at similar elution volumes indicating preservation of the dimeric state. SEC analysis was performed as described in Methods for thermostability analysis of AdiC variants. **Table S1**. Data collection, processing and refinement statistics. **Table S2**. Selected interactions in the substrate-binding site involving water molecules

## Data Availability

The atomic coordinates have been deposited in the Protein Data Bank, www.pdb.org. (PDB ID code 7O82) [49].
