# Supplementary material for: High-resolution structure of the amino acid transporter AdiC reveals insights into the role of water molecules and networks in oligomerization and substrate binding
Source: BMC Biol. 2021 Aug 30;19:179. doi: 10.1186/s12915-021-01102-4 (PMC8406831; doi:10.1186/s12915-021-01102-4)
Supplement: Supplementary file 1 — Additional file 1: Supplementary Figures and Tables. Fig. S1. Schematic representation of the alternating access mechanism. The major conformational changes and states of the transporter are shown, which are necessary to allow alternating substrate access from either side of the membrane to the substrate-binding site. Transporter, substrate and lipid molecules are colored in blue, orange and light brown, respectively. The different conformational states shown are a: outward-open, substrate-free; b: outward-open, substrate-bound; c: outward-facing, substrate occluded; d: substrate-bound, fully occluded; e: inward-facing, substrate occluded; f: inward-open, substrate-bound; g: inward-open, substrate-free; h: substrate-free, fully occluded. Fig. S2. Quality of the electron density of AdiC. All TMs are displayed as viewed from the membrane plane and boxed into TM-groups belonging to inverted repeats (left) and the dimerization interface (right). Starting and ending amino acid residues of corresponding TMs are labeled. The TMs are displayed as sticks (cyan) and the corresponding 2Fo-Fc electron density map is contoured at 1.0 σ and shown as blue colored mesh. Fig. S3. Structural details of AdiC. (A) TM1 and TM6 are discontinuous and their loop regions connecting the α-helical segments are in close proximity and involved in substrate-binding. TM1 and TM6 are represented as light blue ribbons and the side chains as lines. The α-helical segments of TM1 (TM1a and TM1b) and TM6 (TM6a and TM6b) are highlighted as cylinders and labeled accordingly. The respective N- and C-terminal residues of these segments are labeled as well. (B) The largest interface contribution for AdiC homodimer formation is the interaction between TM11 and TM12. TM12 of monomer A (blue) and TM11 of monomer B (yellow) are displayed as ribbons. Interdigitating, non-polar amino acid residues involved in dimer formation are shown as sticks and labeled. Fig. S4. Comparison of the number of water molecules fou [file 12915_2021_1102_MOESM1_ESM.docx]

**Additional file 1** for:

**High-resolution structure of the amino acid transporter AdiC reveals insights into the role of water molecules and networks in oligomerization and substrate binding**

**Hüseyin Ilgü^1^**†**, Jean-Marc Jeckelmann^1^**†**, David Kalbermatter^1^, Zöhre Ucurum^1^, Thomas Lemmin^2,3*^ and Dimitrios Fotiadis^1*^**

^1^ Institute of Biochemistry and Molecular Medicine, and Swiss National Centre of Competence in Research (NCCR) TransCure, University of Bern, CH-3012 Bern, Switzerland

^2^ DS3Lab, System Group, Department of Computer Sciences, ETH Zurich, CH-8093 Zürich, Switzerland

^3^ Trkola Group, Institute for Medicinal Virology, University of Zurich, CH-8057 Zürich, Switzerland

† Hüseyin Ilgü and Jean-Marc Jeckelmann contributed equally to this work.

^*^ Correspondence: [thomas.lemmin@inf.ethz.ch](mailto:thomas.lemmin@inf.ethz.ch); [dimitrios.fotiadis@ibmm.unibe.ch](mailto:dimitrios.fotiadis@ibmm.unibe.ch)

**Fig. S1**


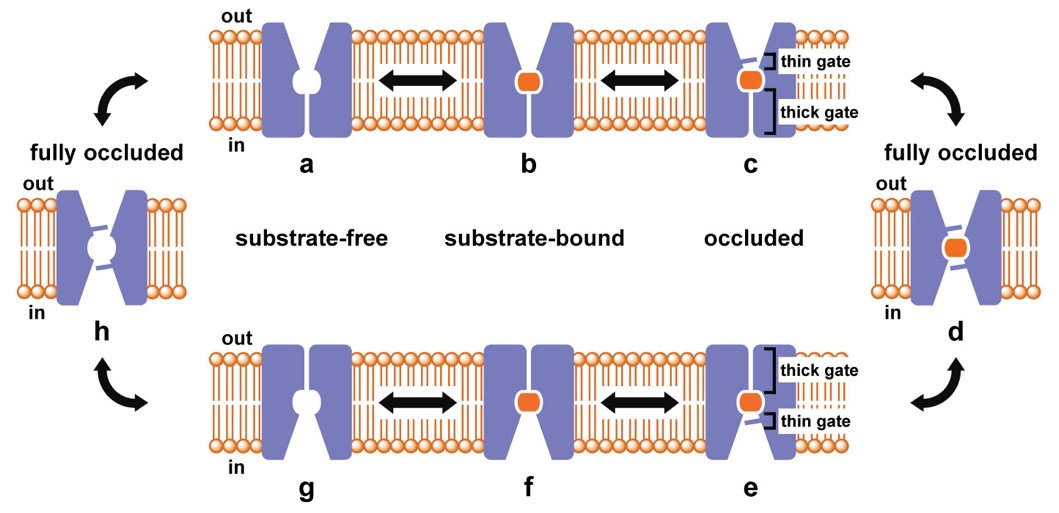


**Fig. S2**


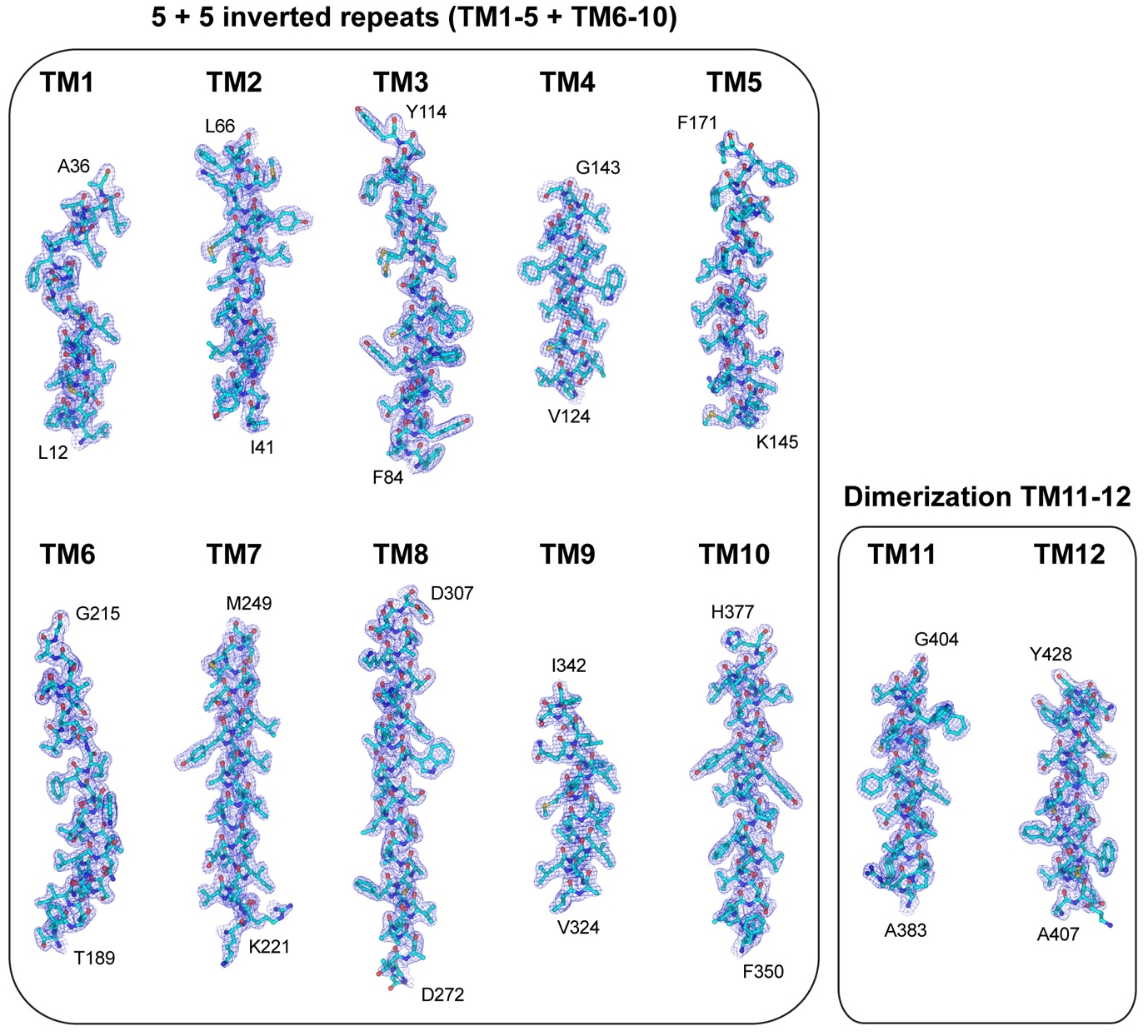


**Fig. S3**


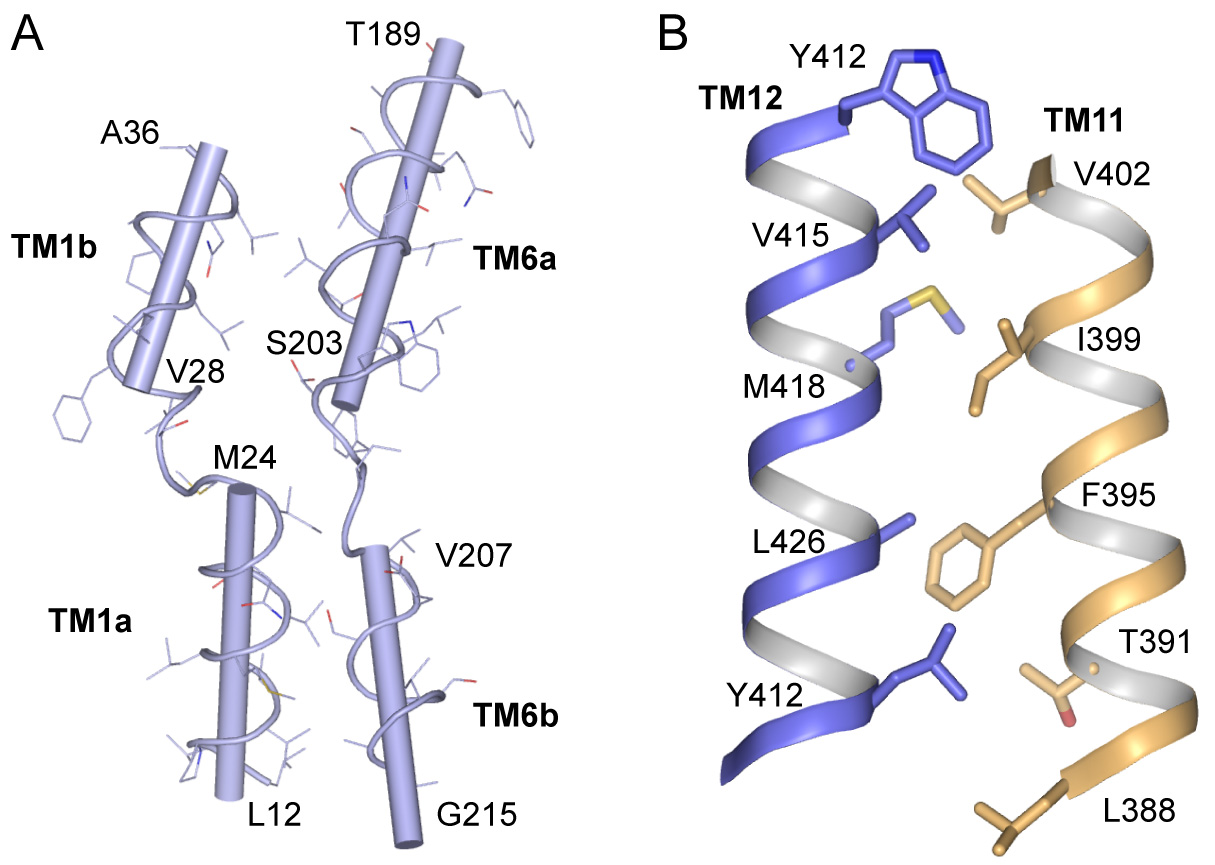


**Fig. S4**


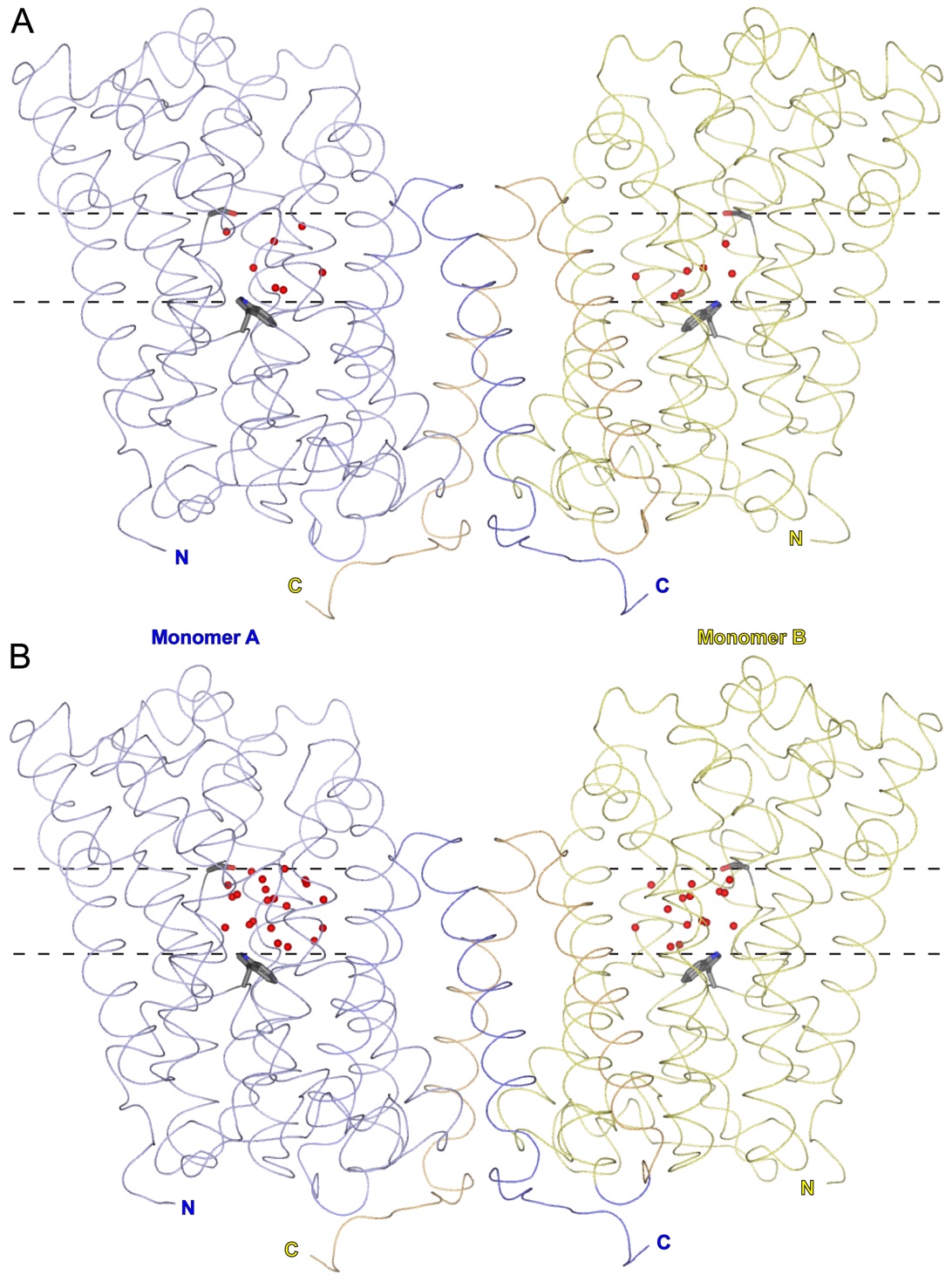


**Fig. S5**


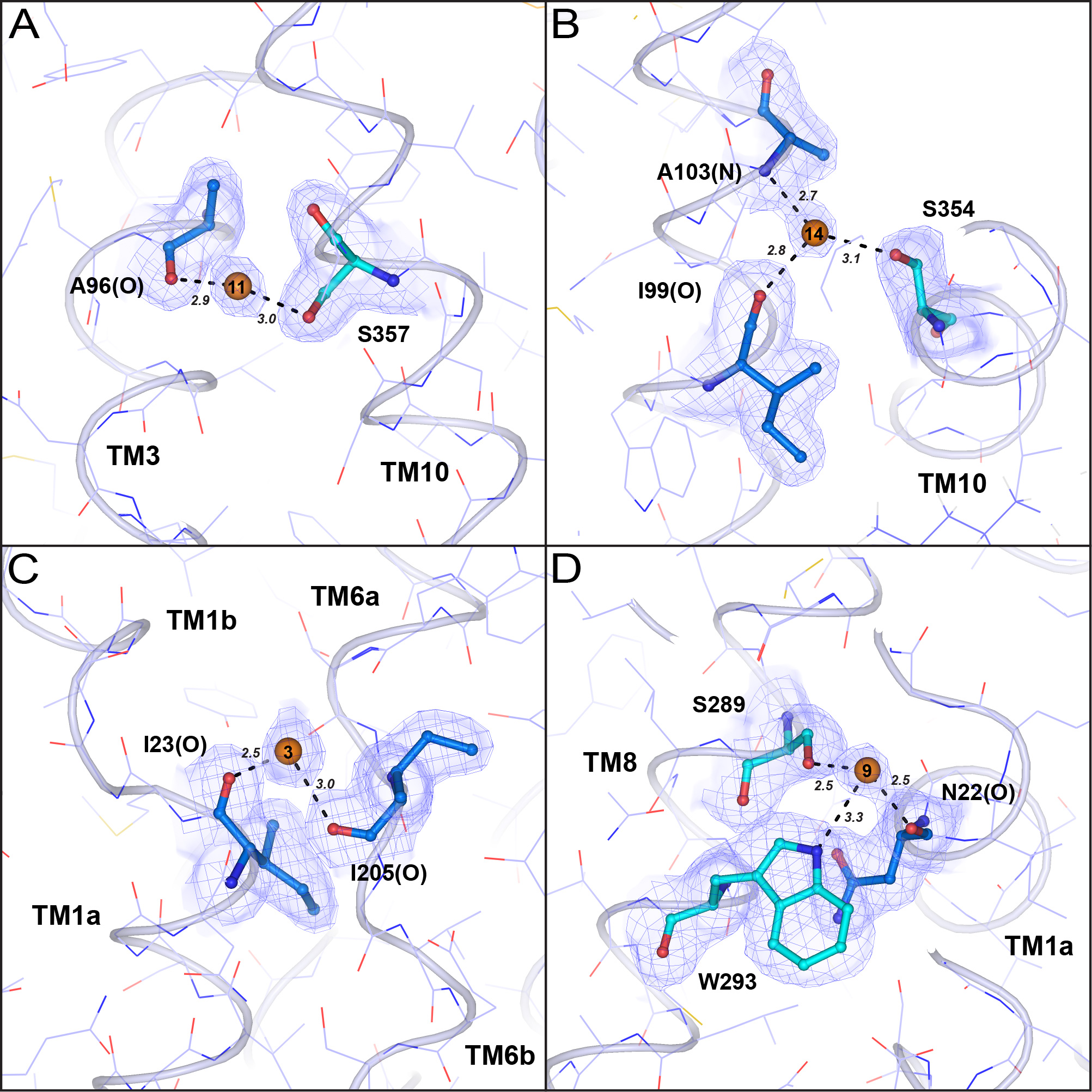


**Fig. S6**

**
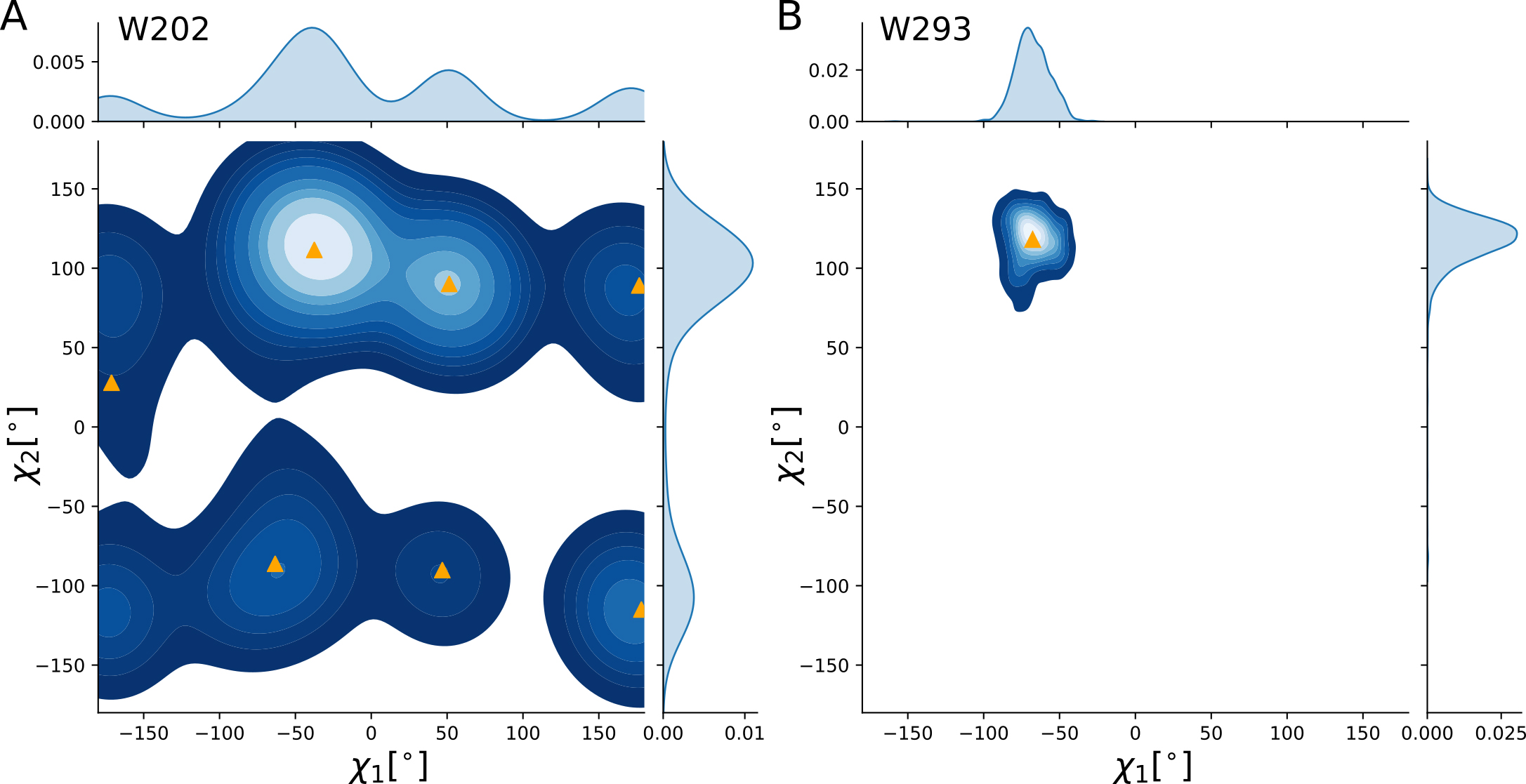
**

**Fig. S7**


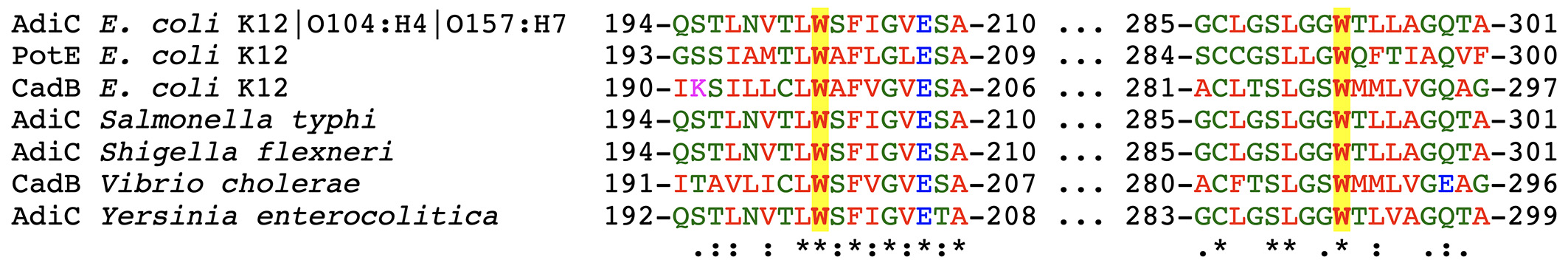


**Fig. S8**


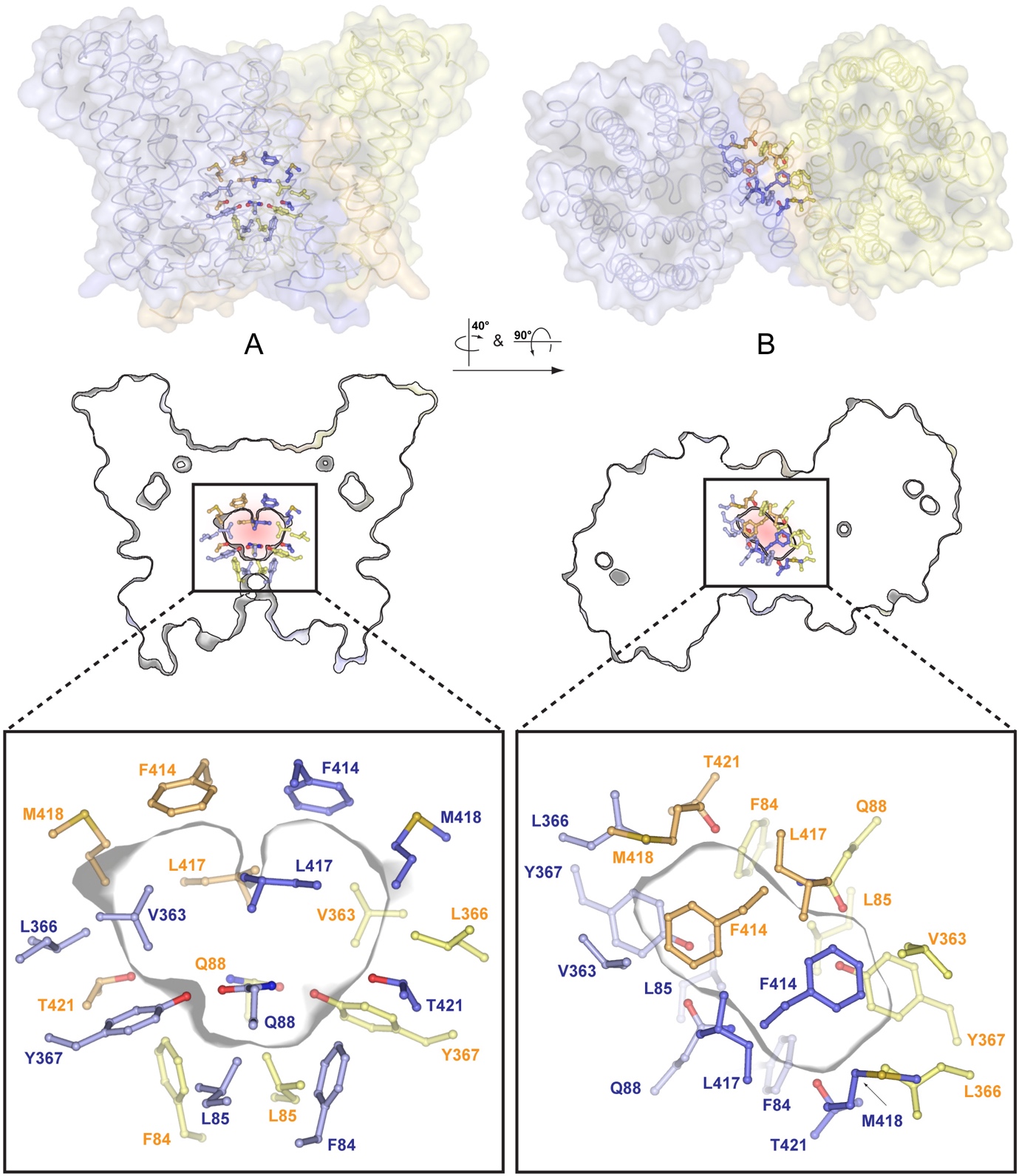


**Fig. S9**


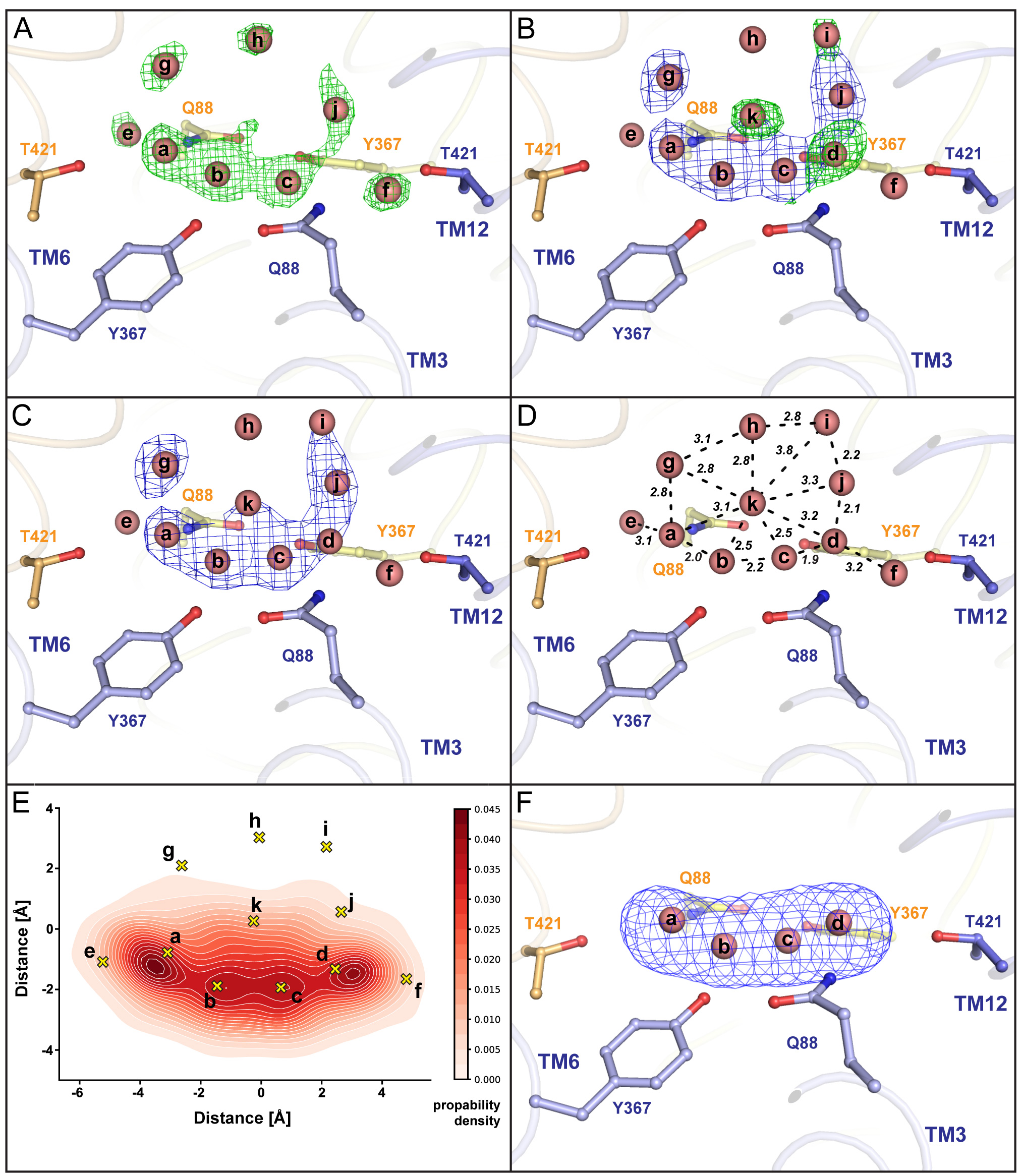


**Fig. S10**


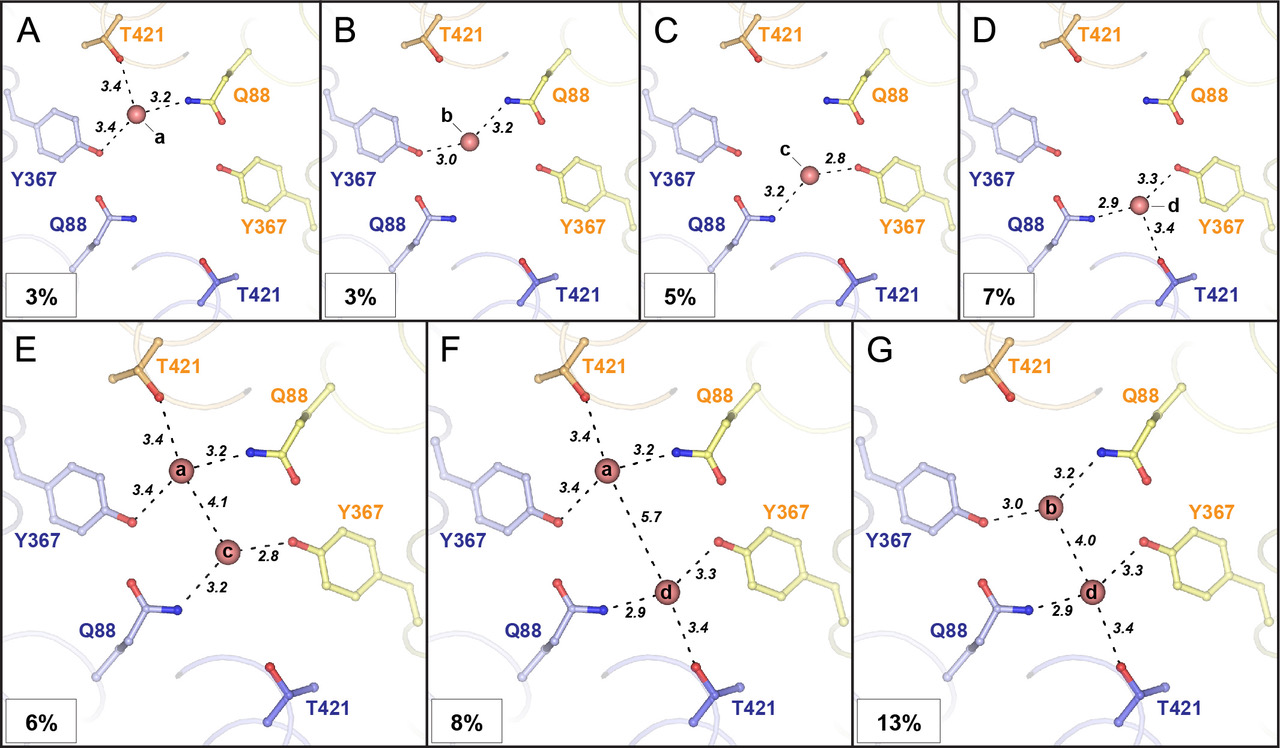


**Fig. S11**


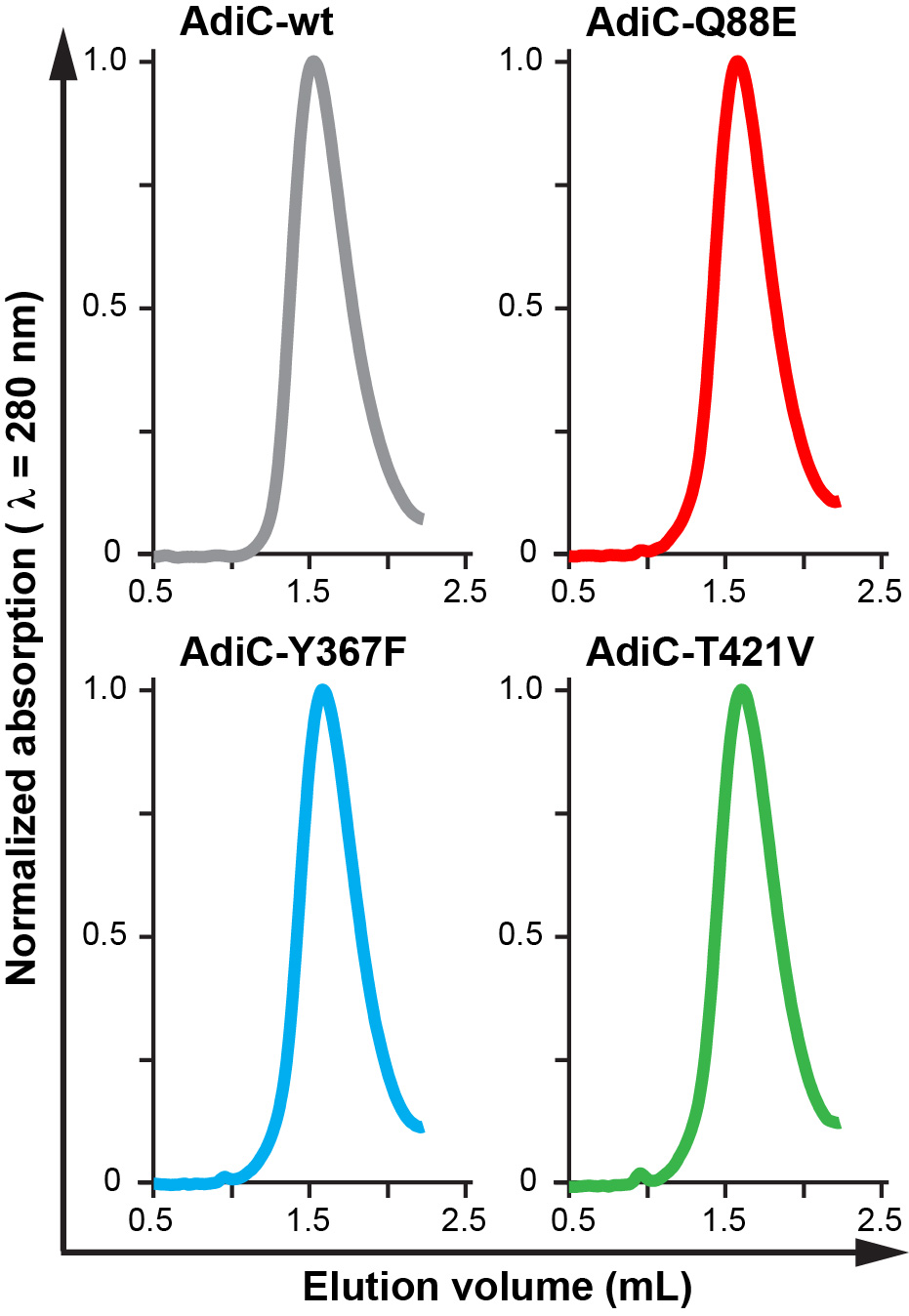


| **Table S1. Data collection, processing and refinement statistics** | | |
| --- | --- | --- |
| **Data collection^a^ and processing** |  | |
| Beamline | X06SA, Swiss Light Source - SLS | |
| Detector | Eiger 16M | |
| Space group | *P*2_1_2_1_2 | |
| Unit-cell: *a*, *b*, *c* (Å); *α = β =* *γ* (°) | 104.7, 175.6, 73.3; 90 | |
| Anisotropy direction^b^ | | |
| overall (Å) | **1.69** | |
| along h axis (Å) | 1.95 | |
| along k axis (Å) | 1.73 | |
| along l axis (Å) | 1.69 | |
| Resolution (Å)^c,d^ | 56.81-1.69 (1.79-1.69) | |
| Measured reflections | 14,403,674 (541,101) | |
| Unique reflections | 118,568 (5,173) | |
| Redundancy | 121.5 (104.6) | |
| *R*_meas_^e^ | 0.18 (7.7) | |
| *R*_p.i.m._^f^ | 0.02 (0.7) | |
| CC_1/2_^g^ | 99.2 (57.2) | |
| Mean *I*/*σ*(*I*) | 52.2 (1.4) | |
| Completeness (%)^h^ | 96.4 (84.4) | |
| **Refinement** |  |  |
| Resolution (Å) | 34.59-1.69 | |
| *R*_work_/*R*_free_^i^ (%) | 19.5 / 20.3 | |
| No. of atoms | 7053 | |
| Protein | 6553  143 | |
| Ligand | 143 | |
| Water | 357 | |
| Mean *B* factor (Å^2^) | 51.9 | |
| Protein | 50.4 | |
| Ligand | 90.2 | |
| Water | 59.4 | |
| RMSD |  | |
| Bond length (Å) | 0.012 | |
| Bond angle (°) | 1.216 | |
| Ramachandran plot (%) |  | |
| Favored region | 99.2 | |
| Allowed region | 0.8 | |
| Disallowed region | 0 | |

^a^ Datasets from 22 crystals were merged.

^b^ The anisotropic resolution limits were computed with AIMLESS [1]⁠ based on CC_1/2_ > 0.30.

^c^ These statistics are for data that was truncated by STARANISO software (http://staraniso.globalphasing.org/) to remove poorly measured reflections affected by anisotropy.

^d^ Values in parentheses are for the highest resolution shell.

^e^ *R*_meas_ as defined by Diederichs and Karplus (1997) [2].

^f^ Precision-indicating merging *R* factor *R*_p.i.m_ as defined by Weiss (2001) [3].

^g^ CC_1/2_ is the Pearson correlation coefficient of two-half data sets as described by Karplus and Diederichs (2012) [4].

^h^ The completeness after the anisotropic correction was obtained by least-square fitting an ellipsoid to the reciprocal lattice points at the cut-off surface defined by a local mean *I/σI* threshold of 1.2, rejecting outliers in the fit due to spurious deviations, and calculating the fraction of observed data lying inside the ellipsoid.

^i^ Random 5% reflections from working set were excluded from refinement for *R*_free_ calculation.

**Table S2. Selected interactions in the substrate-binding site involving water molecules**

| Linked TM domains | Bridging water molecule | Amino acid residues interacting with water molecule | Interaction(s) displayed in Additional file 1: Fig. S5, panel: | Interaction observed in % of sampled MD conformations* |
| --- | --- | --- | --- | --- |
| TM3 - TM10 | H_2_011 | A96(O) & S357 | A | 37±12% |
| TM3 - TM10 | H_2_014 | I99(O), A103(N) & S354 | B | ** |
| TM1a - TM6 | H_2_03 | I23(O) & I205(O) | C | 73±9% |
| TM1a - TM8 | H_2_09 | N22(O), S289 & W293 | D | 25±5% |

* Mean±standard deviation

** Interaction rarely observed, because I99(O) preferred forming a direct hydrogen bond with A103(N) in MD simulations.

**References**

1. Evans PR, Murshudov GN. How good are my data and what is the resolution? Acta Crystallogr D Biol Crystallogr. 2013;69(7):1204-14. <https://doi.org/10.1107/S0907444913000061>.

2. Diederichs K, Karplus PA. Improved R-factors for diffraction data analysis in macromolecular crystallography. Nat Struct Biol. 1997;4(4):269-75. <https://doi.org/10.1038/nsb0497-269>.

3. Weiss MS. Global indicators of X-ray data quality. J Appl Cryst 2001;34:130-5. <https://doi.org/10.1107/S0021889800018227>.

4. Karplus PA, Diederichs K. Linking crystallographic model and data quality. Science. 2012;336(6084):1030-3. <https://doi.org/10.1126/science.1218231>.
